# Controlled Growth and Bandstructure Properties of One Dimensional Cadmium Sulfide Nanorods for Visible Photocatalytic Hydrogen Evolution Reaction

**DOI:** 10.3390/nano10040619

**Published:** 2020-03-27

**Authors:** Rama Krishna Chava, Namgyu Son, Yang Soo Kim, Misook Kang

**Affiliations:** 1 Department of Chemistry, College of Natural Science, Yeungnam University, 280 Daehak-Ro, Gyeongsan, Gyeongbuk 38541, Korea; sng1107@ynu.ac.kr; 2 Korea Basic Science Institute, Gwahak-ro, Yuseong-gu, Daejeon 34133, Korea; kimyangsoo@kbsi.re.kr

**Keywords:** CdS nanorods, solvothermal synthesis, bandstructure, photocatalytic H_2_ evolution, electron-hole recombination

## Abstract

One dimensional (1D) metal sulfide nanostructures are one of the most promising materials for photocatalytic water splitting reactions to produce hydrogen (H_2_). However, tuning the nanostructural, optical, electrical and chemical properties of metal sulfides is a challenging task for the fabrication of highly efficient photocatalysts. Herein, 1D CdS nanorods (NRs) were synthesized by a facile and low-cost solvothermal method, in which reaction time played a significant role for increasing the length of CdS NRs from 100 nm to several micrometers. It is confirmed that as the length of CdS NR increases, the visible photocatalytic H_2_ evolution activity also increases and the CdS NR sample obtained at 18 hr. reaction time exhibited the highest H_2_ evolution activity of 206.07 μmol.g^−1^.h^−1^. The higher H_2_ evolution activity is explained by the improved optical absorption properties, enhanced electronic bandstructure and decreased electron-hole recombination rate.

## 1. Introduction

The yearning for renewable and sustainable energy, as well as environmental protection, has initiated tremendous research interest in photocatalysis, in which sunlight is used as a primary energy source. The photocatalytic water splitting reaction is a promising approach to convert the solar energy into high energy density fuel hydrogen (H_2_) to solve energy and environmental issues [1,2,3]. A suitable semiconductor, be it organic or inorganic material, is simply needed to bring about the desirable photo(electro)-chemical reaction. Apart from being cost-effective and environmentally benign, the practicability of a semiconductor for targeted applications mostly relies on the solar energy conversion efficiency of the material [4,5,6]. Since Fujishima-Honda reported photoelectrochemical water splitting [7], numerous photocatalysts, such as TiO_2_ [8,9], g-C_3_N_4_ [10,11], MoS_2_ [12,13], ZnIn_2_S_4_ [14,15], etc., have been extensively studied for their viability and efficiency in the photocatalyst field. Alternatively, substantial efforts have been made for the development of visible-light-driven photocatalysts, capable of absorbing visible light (≥420 nm) which is about 43% of the solar spectrum. Metal sulfides are considered as good candidates and among the various metal sulfide semiconductor photocatalysts, cadmium sulfide (CdS) has been widely explored as a visible light photocatalyst due to its narrow bandgap, proper bandstructures and higher negative conduction band edge position than H^+^/H_2_ redox potential [16,17]. However, the ultrafast charge recombination rate and fewer active sites on the surface of CdS yield low photocatalytic activity, hence considerably limiting the applications of CdS in H_2_ evolution reactions. As the H_2_ evolution activity of CdS materials continue to increase, several CdS nanostructures have been proposed for the efficient photocatalytic H_2_ evolution reactions [18,19,20,21,22,23]. It is testified that the photocatalytic H_2_ evolution activity of CdS greatly depends on its crystal structure, morphology, crystallinity and size [24], and all these parameters directly impact the band structures, bandgap energy and further electron-hole separation processes. Therefore, the design and synthesis of CdS nanostructures with higher surface areas, charge separation efficiency and with abundant active sites are indeed important.

Meanwhile, one dimensional (1D) CdS nanostructures have received particular attention for the visible photocatalytic H_2_ evolution reactions due to their distinctive geometrical and electronic properties, which can provide large aspect-ratio, direct pathways for charge transport and decouple the direction of charge carrier collection [25,26]. In addition to the fast and long-distance photoexcited electron transport, high length-to-diameter of 1D nanostructures could improve the light absorption and scattering, which benefits the photocatalysis [27,28,29,30,31]. As a result, 1D nanostructures offer a suitable platform for understanding fundamental concepts about the roles of dimensionality and size in optical, electrical, and for photocatalytic solar energy conversion applications [27,32]. Among the several methods for the preparation of 1D CdS, the solvothermal approach is one of the ideal methods due to its simplicity, low cost, scalability, possibilities for the precise control of nucleation/growth, crystal structure, size and morphology [33]. Mostly, 1D CdS nanorods were synthesized by using ethylenediamine as solvent, for example, Jang et al utilized the solvothermal approach and obtained CdS nanowires at different reaction temperatures and times [34]. Next, Li et al. synthesized CdS nanorods (NRs) by using the hydrothermal method in which ethylenediamine acts as the template and coordination agent [35]. The results showed that the photocatalytic H_2_ evolution activities of CdS samples depend on the crystallinity, morphology and surface area. However, the quality of produced CdS NRs in the above reports is very poor since the final product contains a mixture of nanoparticles, nanosheets and nanorods instead of a single morphology. These types of products impede the photogenerated charge carriers’ separation and electron transfer process, consequently producing lower photocatalytic activity. Thus, it is highly desirable to produce high quality 1D CdS NRS for efficient photocatalytic H_2_ evolution reactions.

Herein, we report the modified solvothermal synthesis of 1D CdS NRs at different reaction times by using elthylenediamine as solvent (at a temperature of 180 °C). The reaction time played a significant role in increasing the length of CdS NRs from 100 nm to several micrometers. The band structures, optical properties, and specific surface area of CdS NRs were determined. Moreover, the effect of CdS NR length on the photocatalytic H_2_ evolution activity was systematically investigated.

## 2. Materials and Methods 

### 2.1. Materials

Cadmium nitrate (Cd(NO_3_)_2_.4H_2_O), ethylene diamine (C_2_H_8_N_2_), Thiourea (CH_4_N_2_S), lactic acid (C_3_H_6_O_3_), and triethanol amine (C_6_H_15_NO_3_) were purchased from Daejung Chemicals Co., Ltd. All chemicals were used directly without any further purification. Double distilled (DI) water was used for solution making purposes throughout the experiments.

### 2.2. Synthesis of CdS Nanorods (CdS NRs)

CdS nanorods with different sizes were prepared by a solvothermal reaction at a fixed reaction temperature of 180 °C. In brief, cadmium nitrate (1.92 g) and thiourea (1.42 g) were dissolved in ethylenediamine (EDA) and stirred for 15 min. After stirring, the clear solution was transferred to a 100 mL Teflon lined stainless steel autoclave and heated at 180 °C for 24 h. After cooling down to room temperature, the yellow colored precipitates were washed with ethanol and DI water several times and dried at 70 °C overnight. The obtained sample was named as CdS-24. The other samples were prepared in a similar manner at the reaction times of 6, 12, 18, and 48 h and the corresponding samples were named as C-6, C-12, C-18, and C-48 respectively.

### 2.3. Characterization Methods

Field emission-scanning electron microscope (FE-SEM) images were recorded on a Hitachi S-4800 (Hitachi, Japan). Transmission electron microscope (TEM) images were procured from a H-7600 instrument (Hitachi, Japan). Powder X-ray diffraction analysis was conducted on a Panalytical XpertPro diffractometer (PANalytical B.V., The Netherlands) with Cu-Kα radiation. Raman spectra of the prepared samples were recorded on a Horiba XPlora Plus spectrophotometer (HORIBA Ltd., Japan) operating with a laser source of 532 nm. Laser light was focused on the sample using a 100× magnification Olympus lens and a 1800-line grating was used along with an accumulation time of 2 s and 20 repetitions to collect the spectrum. Nitrogen adsorption and desorption isotherms of all CdS samples were measured on a BELSorp-II Mini instrument (BEL. Japan), from which the Brunauer–Emmett–Teller (BET) surface area, pore volume, and average pore diameter were determined. UV-vis diffuse reflectance spectra (DRS) of the prepared samples were recorded on a Scinco spectrophotometer (SCINCO Co. Ltd., South Korea) in the range of 300–900 nm. Photoluminescence (PL) spectra were taken from a Scinco spectrofluorometer (SCINCO Co. Ltd., South Korea) at the excitation wavelength of 350 nm. The X-ray photo electron spectroscopy measurements were obtained from a Thermo-Scientific K-alpha instrument (Thermo Fisher Scientific Inc., Waltham, MA, USA).

### 2.4. Photocatalytic H_2_ Evolution Measurements

Photocatalytic activities of all CdS samples were checked toward H_2_ evolution reaction. At first, a 5 mg CdS sample was suspended in a photocatalytic reactor which contained 10% lactic acid aqueous solution. The reactor was closed tightly with a rubber septum and flushed with Ar gas for one hour to remove any dissolved gases present in the system. A visible light source of 150 W was used for irradiation and for every one hour, 200 μL gas was sampled and analyzed by gas chromatography.

## 3. Results and Discussion

### 3.1. Morphological and Structural Studies

**Scheme 1:**
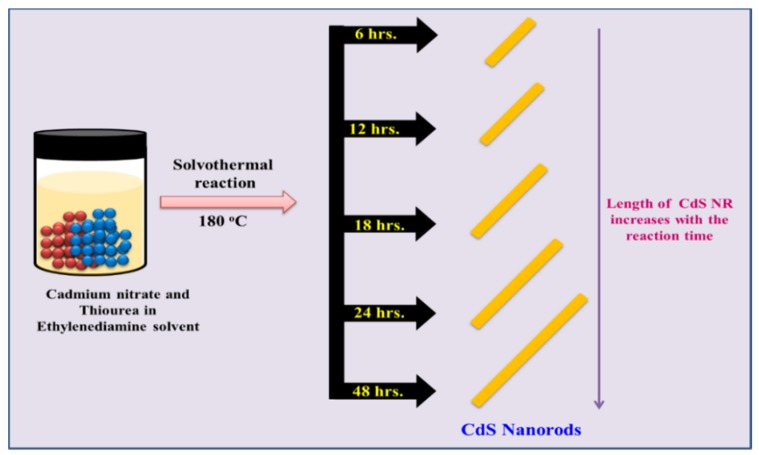
The process for synthesizing the 1D CdS nanorods of different lengths.

**Figure 1:**
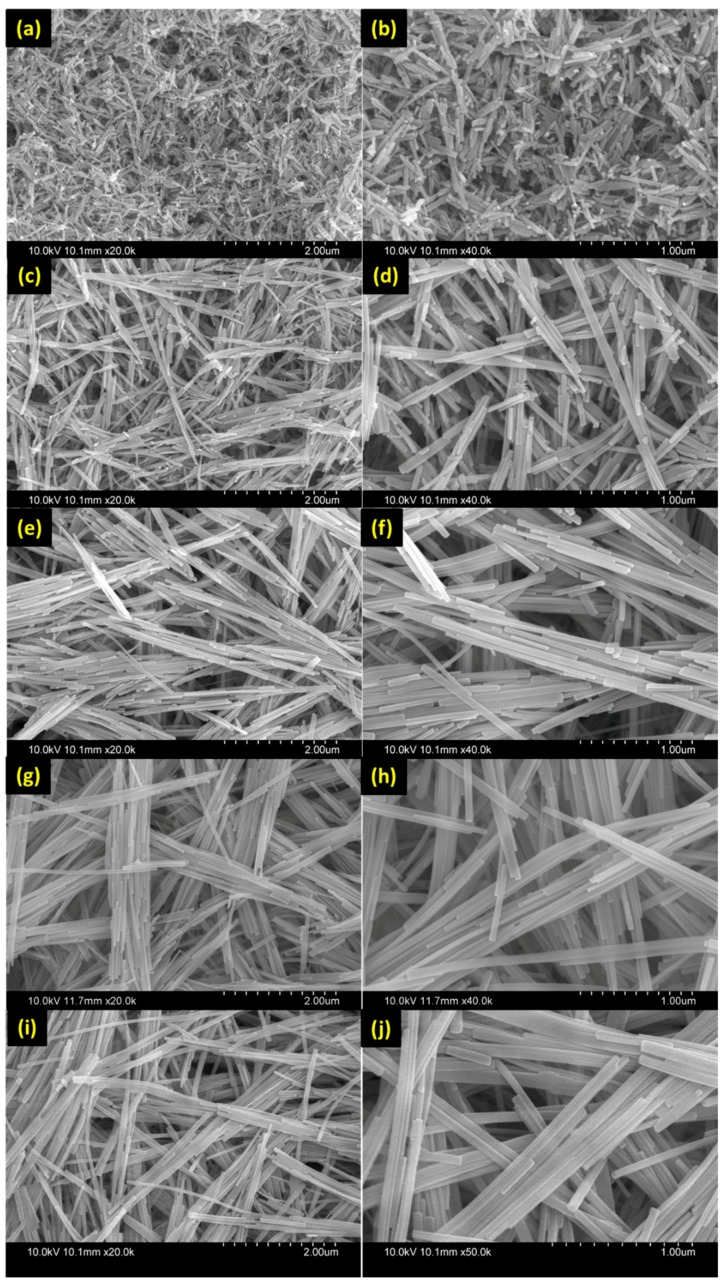
FE-SEM images of as prepared CdS nanorods at a reaction times of 6 h (**a**,**b**), 12 h (**c**,**d**), 18 h (**e**,**f**), 24 h (**g**,**h**), and 48 h (**i**,**j**).

**Figure 2:**
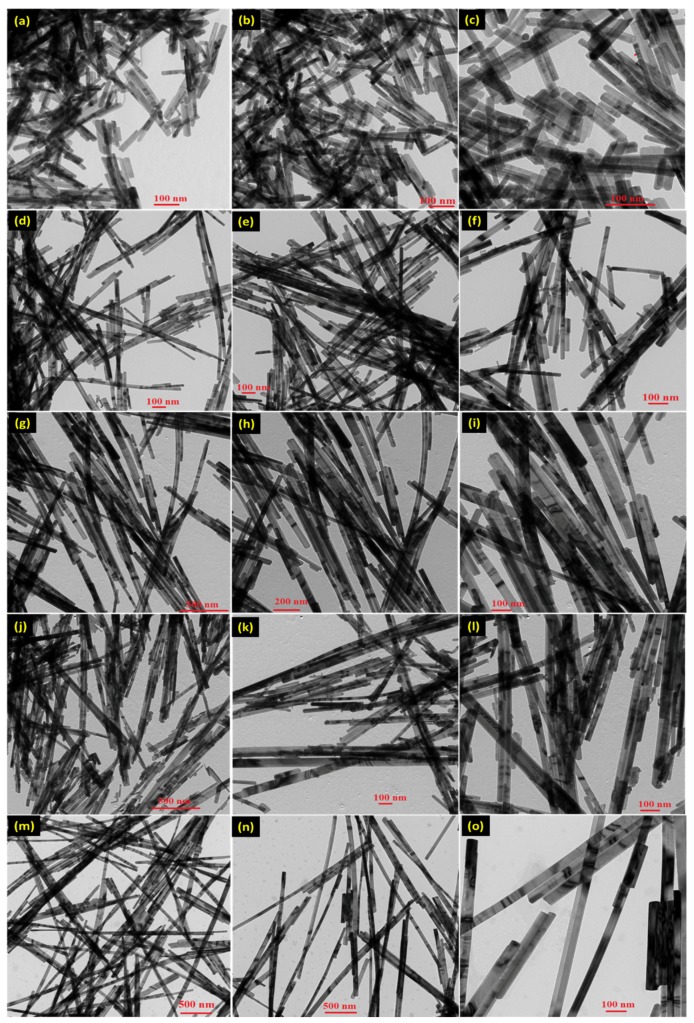
TEM images of as prepared CdS nanorods at reaction times of 6 h (**a**–**c**), 12 h (**d**–**f**), 18 h (**g**–**i**), 24 h (**j**–**l**), and 48 h (**m**–**o**).

CdS nanorods were prepared by using a solvothermal method in which ethylenediamine (EDA) was used as solvent and the detailed process is illustrated in Scheme 1. The FE-SEM images of prepared CdS nanorods at different reaction times are displayed in Figure 1. According to Figure 1, the obtained CdS sample at all reaction times exhibits the nanorod morphology. As seen from FE-SEM images, the length of nanorods increases with the reaction time. Moreover at lower reaction times (6, 12 and 18 h), the CdS nanorods sample contains short nanorods with the long nanorods. At reaction temperatures of 24 and 48 h, the purity of the sample is high. Next, to further observe the morphological features, TEM images were also recorded and are shown in Figure 2. As seen from Figure 2, all CdS samples exhibit rod-like morphology with a diameter of 40 nm and as the reaction time increases the length of CdS nanorods increases to several micrometers. From TEM images, the average diameter of as prepared samples was calculated by using the statistical measurements. The observed diameters for the CdS nanorods are 13, 27, 29, 39 and 54 nm for C-6, C-12, C-18, C-24 and C-48 samples respectively. From these measurements, it was confirmed that the diameter of CdS nanorods also increases with the reaction time.

**Figure 3:**
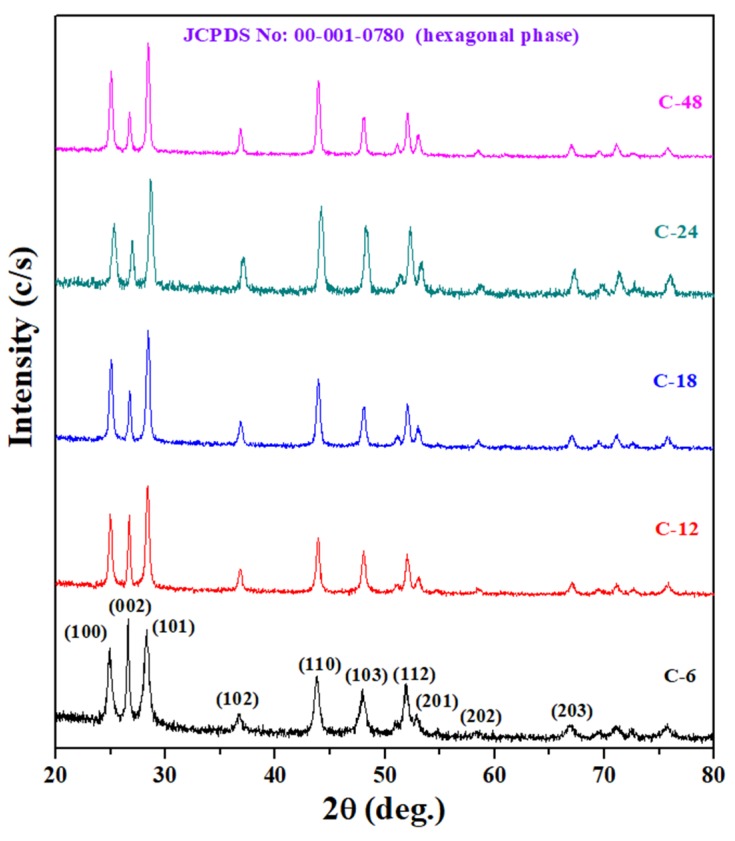
Powder XRD patterns of CdS nanorods obtained at different reaction times.

The crystal structures of synthesized CdS samples were determined by X-ray diffraction studies. As shown in Figure 3, the diffraction patterns of all CdS samples show similar features. The diffraction peaks of CdS nanorods (C-6) were observed at 24.85°, 26.60°, 28.24°, 36.76°, 43.75°, 47.95°, 51.95°, 52.88°, 58.42° and 66.95° can be assigned to the (100), (002), (101), (102), (110), (103), (112), (201), (202) and (203) planes of hexagonal CdS phase (JCPDS No: 00-001-0780) with cell constants of a = b = 0.4142 and c = 0.6724 nm. The crystallinity is good for all samples. As seen from Figure 3, the intensity of the (002) plane is observed to be high when compared to (100) and (101) planes for sample C-6, however as the reaction time increases the intensity of (100), and (101) planes increases while that of the (002) plane decreases, which may be a result of the growth of CdS nanorods to several micrometers.

**Figure 4:**
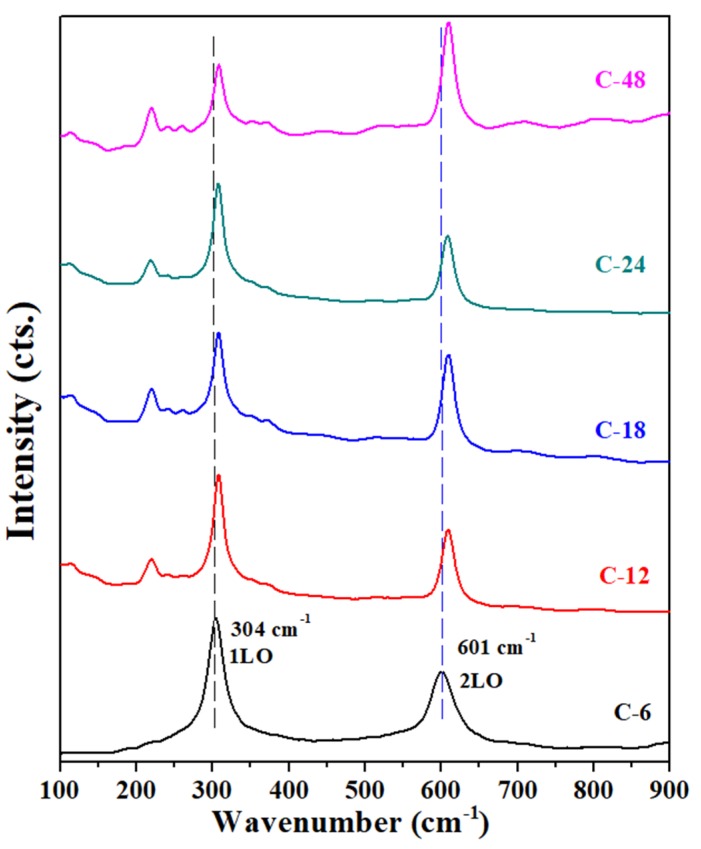
Raman spectra of CdS nanorods prepared at different reaction times.

The structural properties of the synthesized CdS NR samples were also studied by Raman spectroscopy. Since the CdS NRs belong to a hexagonal wurtzite structure with a space group of C_6_^4v^, according to group theory, the vibrational modes 1A_1_ + 1E_1_ + 2E_2_ (E_2H_ and E_2L_) are Raman active and the 2B_2_ modes are silent. The phonon vibrations E_1_ and E_2_ are in the x-y plane whereas the A_1_ phonon modes are in the z-direction [36,37]. The Raman spectra of the prepared CdS nanorods samples are given in Figure 4. As shown in Figure 4, the CdS NR sample C-6 exhibits two bands at 304 and 601 cm^-1^ which are assigned to the first and second order longitudinal optical modes (1-LO and 2-LO modes) respectively [38,39]. As the reaction time increases from 6 to 48 hours, the additional peaks observed below 300 cm^-1^ correspond to the high crystallinity of the samples at higher reaction times consistent with the powder XRD results [40]. The Raman bands of CdS NR samples C-12, C-18, C-24, and C-48 are slightly redshifted with respect to the C-6 sample, which can be ascribed to the lattice expansion along the c-axis since the peak positions are very sensitive to the lattice strain along the c-axis [39,41]. Thus, Raman spectra further confirms the formation CdS nanorods. 

**Figure 5:**
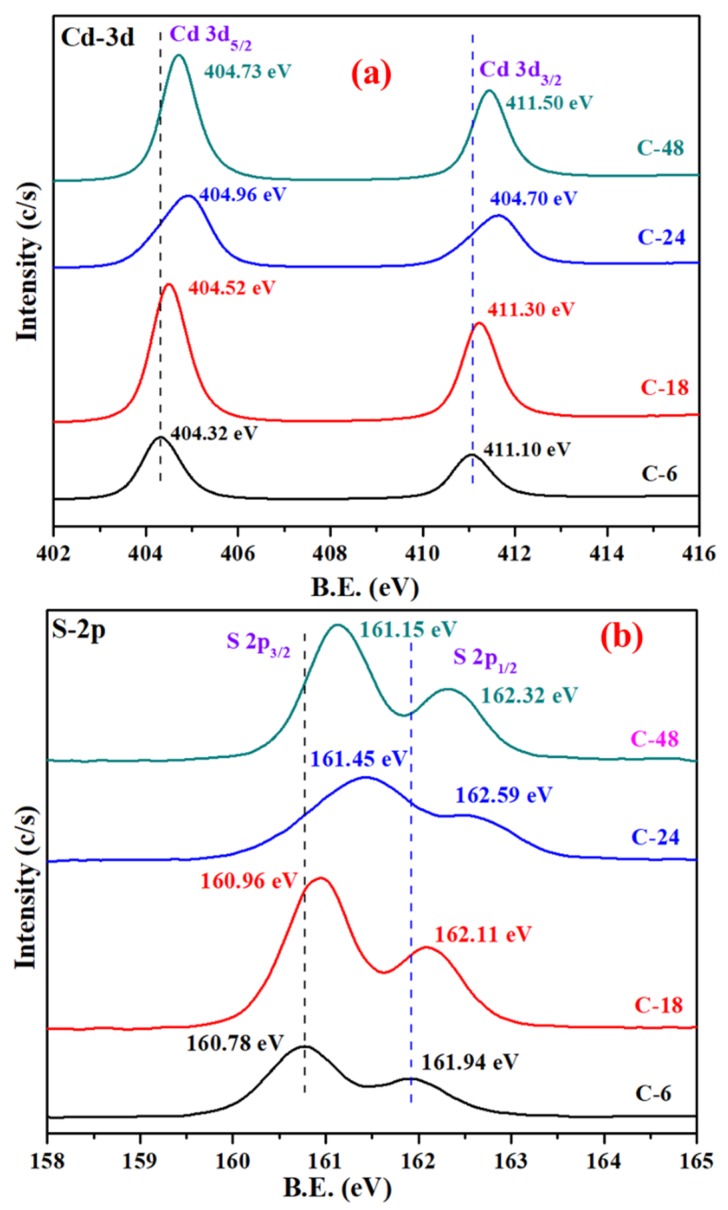
High resolution XPS spectra of (**a**) Cd-3d and (**b**) S-2p of CdS nanorods prepared at different reaction times.

To further analyze the surface and chemical states of prepared CdS samples, XPS measurements were carried out and are shown in Figure 5. As seen from Figure 5a, the high resolution XPS spectrum of Cd-3d core-level exhibit two peaks at 404.32 and 411.10 eV for the C-6 sample. These two binding energy peaks separated by ~6.8 eV are ascribed to Cd-3d_5/2_ and Cd-3d_3/2_ for Cd^2+^ in CdS [42,43]. Next Figure 5b shows the XPS spectrum of S-2p core level which displayed two binding energy peaks around 160.78 and 161.94 eV corresponding to the S-2p_3/2_ and S-2p_1/2_ respectively. The two binding energy peaks separated by 1.16 eV is characteristic of S^2-^ in CdS structure [44,45]. Figure 5a,b also shows the XPS spectra of C-18, C-24 and C-48 samples. The spectra show similar features to that of C-6, however there is a positive shift in the binding energy values as the reaction time increases. Among the samples, C-24 shows the highest positive shift in both Cd-3d and S-2p core-level spectra. From the XPS data and XRD measurements, CdS NRs were successfully prepared. 

**Figure 6:**
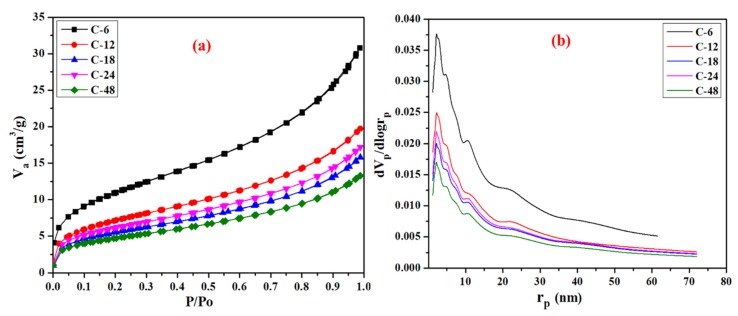
N_2_ adsorption-desorption isotherms (**a**) and pore-size distribution curves (**b**) of CdS nanorod samples prepared at different reaction times.

The specific surface area and pore-size distribution analysis of CdS samples was studied by recording the N_2_ adsorption-desorption isotherms. As shown in Figure 6a, the isotherms for all CdS samples exhibited a typical Type-II isotherm patterns with a narrow H3 Hysteresis loop. Figure 6b shows the pore-size distribution curves which confirms the existence of mesopores. Generally, it is supposed that the H3 hysteresis loop is linked to the mesopores through the aggregates of plate-like particles [46]. However, there were no plate-like particles observed in the current work. Thus, the mesopores may be derived from the random aggregation of CdS NRs, which were further observed from the corresponding pore size distribution curves [47]. Next, the specific surface area and pore-size analysis was performed by using BET and BJH methods and their corresponding parameters were given in Table 1. According to Table 1, the BET surface area of CdS samples were determined as 39, 26, 19, 22 and 16 m^2^/g for C-6, C-12, C-18, C-24 and C-48 respectively.

### 3.2. UV-vis Absorption, Bandgap and Bandstructure Studies

**Figure 7:**
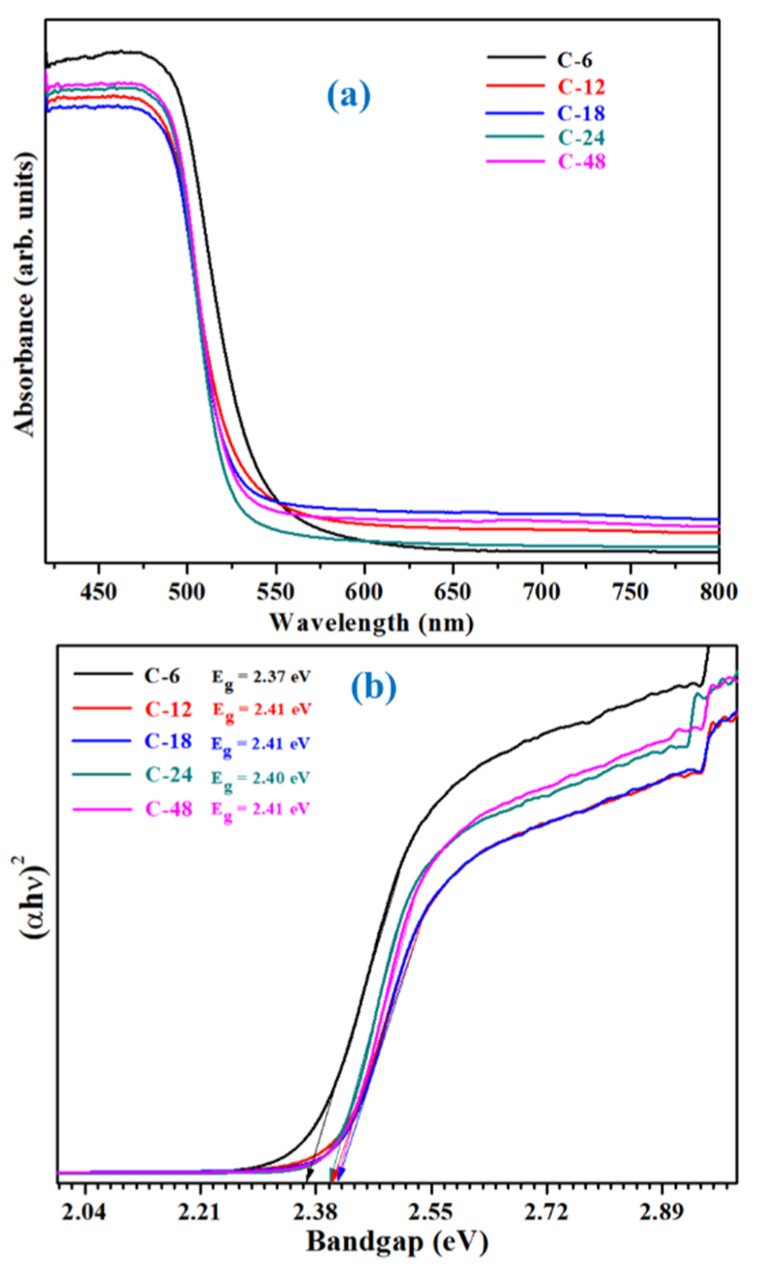
UV-vis diffuse reflectance absorbance spectra (**a**) and (**b**) optical bandgap measurements of CdS nanorods prepared at different reaction times.

The optical absorption properties of the prepared CdS samples were studied by recording the UV-vis absorption spectra and are shown in Figure 7a. As depicted in Figure 7a, all the samples show the absorption edge in the visible region which is consistent with the wurtzite CdS [48,49,50]. The absorption edge of the C-6 sample was observed at 555 nm and as the length of CdS NRs increases, the absorption edge decreases. It is also observed that the absorption capacity of CdS samples increases with the length of nanorods beyond 550 nm and among them the C-18 sample shows the highest absorption features up to near-infrared (NIR) regions. This phenomenon is attributed to the light scattering effect of 1D nanomaterials [51,52]. Usually the photocatalytic activity is determined by the light harvesting capability. CdS NRs with longer diameters can absorb more light at higher wavelengths and generate more electrons and holes for the photocatalytic reactions. 

Next, the optical bandgaps of CdS NR samples were determined from the following equation [53]:

***α***h***ν*** = A(h***ν***-E_g_)^n/2^ (1)

where *α*, h, *υ*, A and E_g_ are the absorption coefficient, Planck’s constant, frequency of incident light, proportionality constant and optical bandgap energy respectively. The value of n depends on the energy transition in a semiconductor with n = 1 for direct transition and n = 1/2 for indirect transition. Here, CdS is characterized by a strong absorption in the visible region and hence its n value could be taken as 1 throughout the calculations. From the bandgap energy equation, Tauc’s plots were analyzed by extrapolating (*α*h*v*)^2^ versus photon energy (h*v*). The intercept of the tangent to the h*v* axis in a Tauc’s plot can give a good estimation of the band gap energy. Consequently, the band gap measurement values of CdS NRs prepared at different reaction times are presented in Figure 7b and determined as 2.37, 2.41, 2.41, 2.40 and 2.41 eV for C-6, C-12, C-18, C-24 and C-48 respectively. The band gap value of C-6 is slightly lower than that of other samples and is in accordance with the light absorption. These results suggest that the bandgap changes in CdS material could display higher photocatalytic activity. 

**Figure 8:**
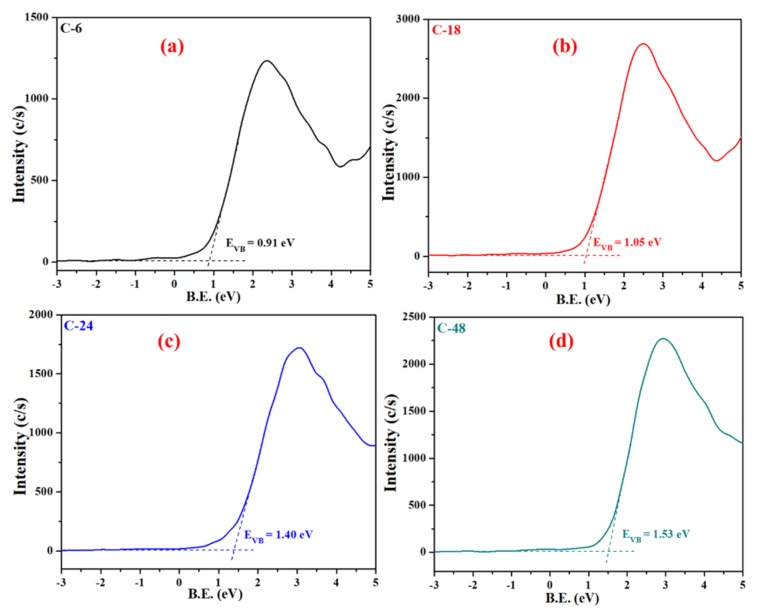
Valence band XPS spectra of CdS NRs prepared at different reaction times: (**a**) C-6, (**b**) C-18, (**c**) C-24, and (**d**) C-48.

In addition, to understand the energy band structures of CdS NRs, valence band (VB) XPS spectra for all CdS samples were recorded and a linear extrapolation was carried out to calculate the VB positions (EVB) of the photocatalysts samples. As seen in Figure 8a–d, the VB positions of C-6, C-18, C-24 and C-48 are 0.91, 1.05, 1.40 and 1.53 eV respectively. Next, the conduction band positions (ECB) of the prepared samples were also calculated by the following equation,

E_CB_ = E_VB_ − E_g_ (2)

Accordingly, the CB positions of C-6, C-18, C-24 and C-48 are calculated as −1.46, −1.36, −1.00 and −0.88 eV respectively. 

**Figure 9:**
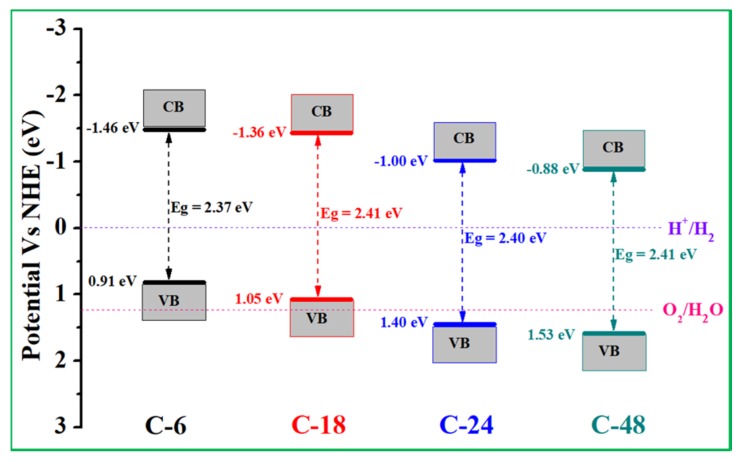
Relative arrangement of valence band (VB) and conduction band (CB) positions for CdS NR samples.

The subsequent band structures of all CdS photocatalyst samples are depicted in Figure 9. According to Figure 9, as the reaction time increases, the VB edge positions are shifted more to the positive whereas the CB edge positions are shifted towards lower reduction potentials.

**Figure 10:**
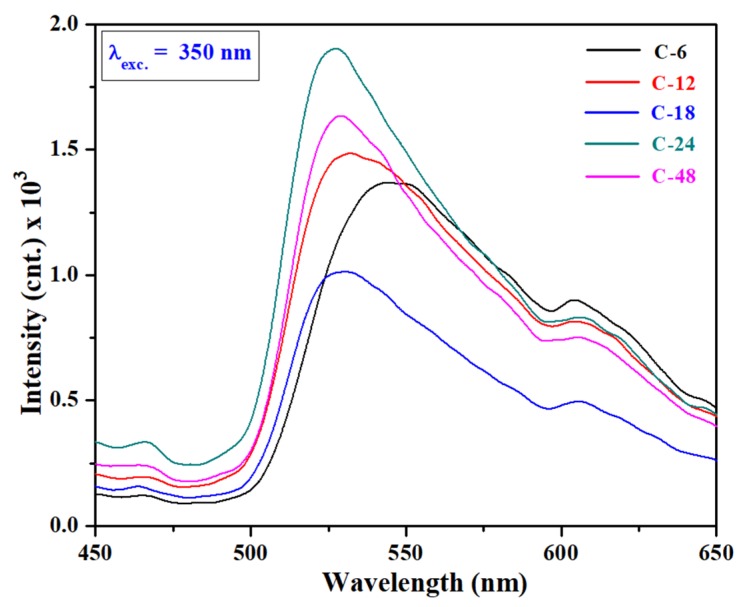
Photoluminescence spectra of CdS nanorods prepared at different reaction times.

Photoluminescence (PL) spectroscopy has been extensively used to understand the defects and photoexcited charge carriers’ separation efficiency which has a direct influence on the photocatalytic activity of semiconductor photocatalysts. It is generally believed that the lower PL intensity signal specifies higher charge separation efficiency. To investigate the electron-hole recombination rate details of CdS NR samples, PL spectra were recorded at the excitation wavelength of 350 nm. As shown in Figure 10, the PL spectrum of the C-6 sample exhibited weak emission peaks at 465 and 605 nm, and an intense emission peak around 545 nm. The two weak peaks at 465 and 605 are attributed to the exciton recombination, defect state emissions and S^2-^ related deep trap emission respectively. The intense emission peak at 545 nm is characteristic of band-band transition [54,55,56,57,58]. In addition, the PL spectra of remaining samples are also depicted in Figure 10. The shape of all spectra is identical, however there is a negative shift in the emission peaks of samples as the length of CdS NRs increase. In addition, the PL intensity of CdS NRs increases with the increased length and the C-24 sample exhibited the highest PL intensity resulting in a higher electron-hole recombination rate. In contrast, sample C-18 shows the lowest PL intensity, which demonstrates that the recombination rate was effectively inhibited. Accordingly, sample C-18 could show higher photocatalytic activity.

### 3.3. Visible Photocatalytic H_2_ Evolution Activity and Mechanism

**Figure 11:**
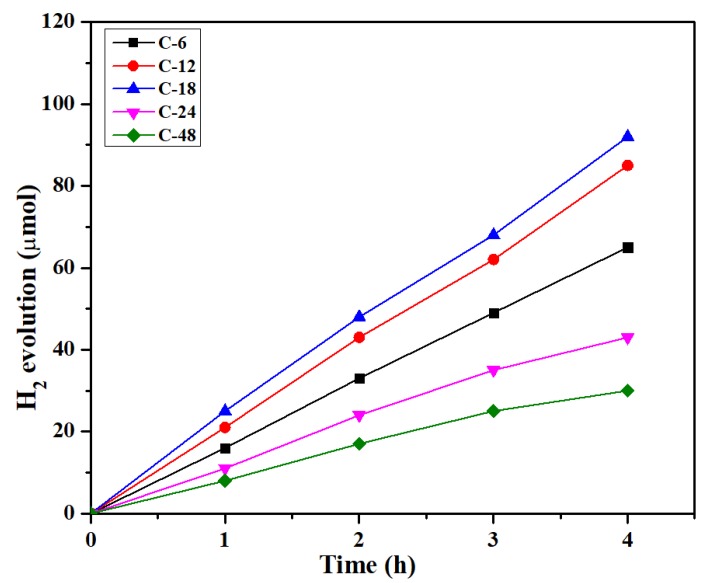
Time course of H_2_ evolution of CdS nanorod samples prepared at different reaction times.

It is reasonable to expect that 1D CdS NRs are a promising kind of photocatalysts due to the fast and long-distance photoexcited electron transport and light scattering properties. Control experiments without irradiation or photocatalysts disclose that no H_2_ evolution was observed. Therefore, the photocatalytic activities of 1D CdS NRs of different lengths were determined by recording the photocatalytic H_2_ evolution under visible light irradiation. In all measurements, 10 vol% lactic acid was used as a sacrificial agent and the reaction was carried out for four hours. The time course measurements show that CdS nanorod samples exhibited a H_2_ evolution activity. The increasing H_2_ production over time (given in Figure 11) specifies the continuous H_2_ production under visible light irradiation. The CdS nanorod sample, C-18 significantly outperforms the other CdS samples. 

**Figure 12:**
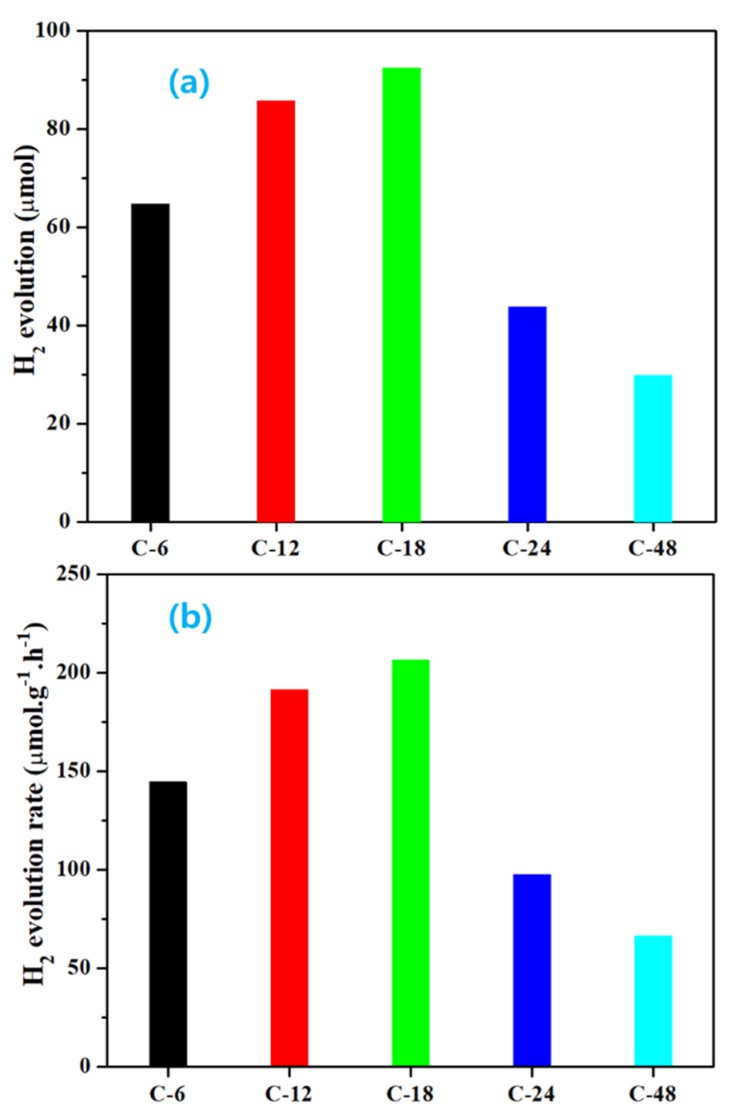
(**a**) H_2_ evolution activities and (**b**) rates of CdS NR photocatalyst samples obtained at different reaction times.

Next, the H_2_ evolution rates of CdS NR samples were shown in Figure 12a. As expected, H_2_ evolution activity of CdS NRs considerably varied with the length. These results showed that the CdS NR sample obtained at a reaction time of 18 hours (C-18) displayed the highest photocatalytic activity. Next, the H_2_ evolution rates of CdS NR samples were measured and presented in Figure 12b. As seen from Figure 12b, C-18 sample exhibited the H_2_ evolution rate of 206 μmol.g^−1^.h^−1^ whereas the remaining samples showed 144, 191, 97 and 66 μmol.g^−1^.h^−1^ for C-6, C-12, C-24 and C-48 respectively. 

In general, a good photocatalyst is characterized by a higher specific surface area since all the photocatalytic reactions occur at the photocatalyst surface. However, in the present work, CdS samples having a higher surface area display a lower photocatalytic activity. Therefore, surface area is not an influential parameter, however, crystallinity played a role in improving the H_2_ evolution activity of CdS samples. As per the XRD patters in Figure 3, the crystallinity of CdS samples increases with the reaction time, indicating that the higher crystallinity sample produces higher photocatalytic activity which is supported by the previous works [18]. Table 2 compares the photocatalytic H_2_ evolution activity of different CdS nanostructures, in which the obtained H_2_ evolution rate in the present work is outperformed and comparable with the reported results.

**Scheme 2:**
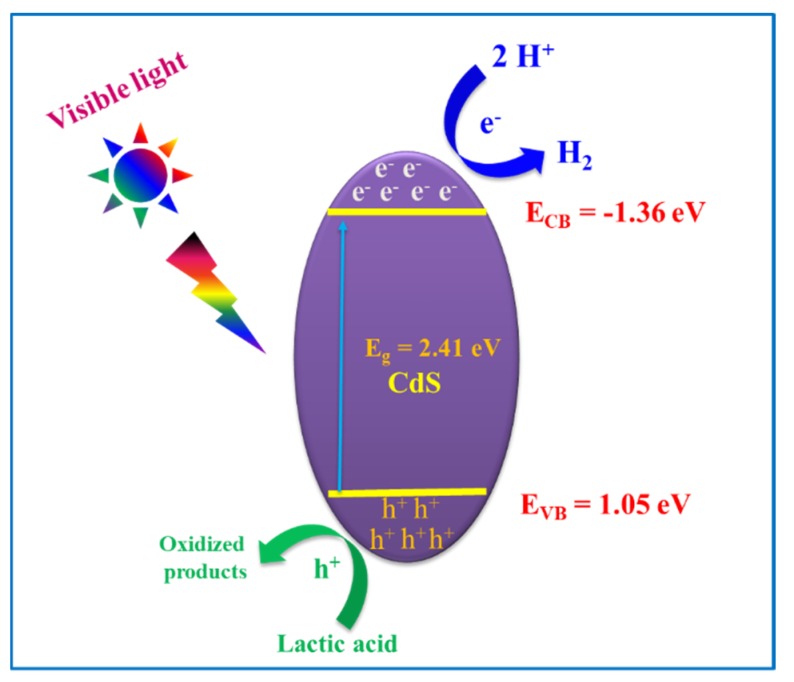
Visible photocatalytic H_2_ evolution mechanism over CdS NR photocatalyst sample.

The schematic illustration for the visible photocatalytic H_2_ evolution activity of the optimized C-18 sample is proposed and depicted in Scheme 2. Generally, the photocatalytic H_2_ evolution activity mainly depends on light absorption properties, band structures and charge separation efficiency. The C-18 sample synthesized in this work possesses optimized length with the improved light absorption properties in the visible region, promoting photocatalytic H_2_ evolution activity. When the photocatalyst sample CdS (C-18) is irradiated with visible light, the electrons in valence band (VB) are excited to conduction band (CB) of CdS and leave holes in the VB. The electrons in the conduction band, having a higher negative redox potential (E_CB_ = −1.36 eV), reduce the 2H^+^ to H_2_. Meanwhile, the holes with positive oxidation potential (E_VB_ = 1.05 eV) in the valence band oxidize the sacrificial agent, lactic acid. Moreover, the suppressed electron-hole recombination rate also promotes the charge separation efficiency (confirmed from PL measurements). As a result, higher photocatalytic H_2_ evolution activity is achieved for the C-18 sample via improved visible light absorption, optimized CdS NR length, higher negative conduction band and lower photoexcited carrier recombination rate.

## 4. Conclusions

In summary, 1D CdS nanorods of different lengths were synthesized by a facile one step solvothermal method, in which ethylenediamine was used as solvent. Experiments show that the reaction time is an essential factor to control the growth of nanorods and the product obtained at an 18 hours reaction time is the optimum for photocatalytic H_2_ evolution reactions. Extensive structural and morphological characterizations of the prepared materials establish that CdS NRs were successfully prepared with suitable electronic band structures. The C-18 sample with distinct properties, exhibited a higher photocatalytic H_2_ evolution activity of 206 μmol.g^−1^.h^−1^ under visible light irradiation. This higher photocatalytic activity of the C-18 sample could be ascribed to higher visible light utilization capacity at the longer wavelengths, more negative conduction band with higher redox potentials and lower photogenerated electron-hole recombination rates. It is anticipated that the present work will improve understanding of the applicability of 1D metal sulfide nanorods for the semiconductor photocatalysts. 

## Figures and Tables

**Table 1:** Surface area and pore-size distribution parameters of CdS NR samples.

Sample	BET Surface Area (m^2^/g)	Pore Volume (cm^3^/g)	Pore Diameter (nm)
C-6	39	0.047	4.8
C-12	26	0.030	4.7
C-18	19	0.024	4.9
C-24	21	0.026	4.8
C-48	16	0.020	4.9

**Table 2:** Comparison of the H_2_ evolution rates of CdS photocatalysts.

Sample	H_2_ Evolution Rate	Reference
CdS nanowires	803 μmol.h^-1^	[18]
CdS hollowstructures	21654 μmol.g^-1^.h^-1^	[19]
CdS nanoflowers	468.7 μmol.h^-1^	[20]
CdS hollowstructures	3.52 mmol.g^−1^.h^−1^	[21]
CdS nanosheets	6.7 mmol.g^−1^.h^−1^	[22]
CdS nanowires	4 μmol.g^−1^.h^−1^	[34]
CdS nanostructures	223 μmol.h^−1^	[35]
CdS petal-like nanostructures	117 μmol.g^−1^.h^−1^	[59]
CdS nanoporous structures	4 μmol.g^−1^.h^−1^	[60]
CdS nanorods	206 μmol.g^−1^.h^−1^	Present work
